# Enhancing Antibody Exposure in the Central Nervous System: Mechanisms of Uptake, Clearance, and Strategies for Improved Brain Delivery

**DOI:** 10.3390/jnt4040020

**Published:** 2023-10-02

**Authors:** Kelly Schwinghamer, Teruna J. Siahaan

**Affiliations:** Department of Pharmaceutical Chemistry, The University of Kansas, Lawrence, KS 66046, USA;

**Keywords:** BBB, BCSFB, mAb brain delivery methods, mAb brain clearance, mAb brain retention, glymphatic system, mechanism of mAb uptake

## Abstract

Antibodies (mAbs) are attractive molecules for their application as a diagnostic and therapeutic agent for diseases of the central nervous system (CNS). mAbs can be generated to have high affinity and specificity to target molecules in the CNS. Unfortunately, only a very small number of mAbs have been specifically developed and approved for neurological indications. This is primarily attributed to their low exposure within the CNS, hindering their ability to reach and effectively engage their potential targets in the brain. This review discusses aspects of various barriers such as the blood–brain barrier (BBB) and blood–cerebrospinal fluid (CSF) barrier (BCSFB) that regulate the entry and clearance of mAbs into and from the brain. The roles of the glymphatic system on brain exposure and clearance are being described. We also discuss the proposed mechanisms of the uptake of mAbs into the brain and for clearance. Finally, several methods of enhancing the exposure of mAbs in the CNS were discussed, including receptor-mediated transcytosis, osmotic BBB opening, focused ultrasound (FUS), BBB-modulating peptides, and enhancement of mAb brain retention.

## 1. Introduction

Monoclonal antibodies (mAbs) have emerged as promising therapeutic and diagnostic candidates for a wide range of diseases due to their ability to target specific molecules with high affinity. They offer advantages including low toxicity, long systemic half-lives, and the capacity for large-scale production with high purity. However, the development of mAbs for central nervous system (CNS) diseases is hampered by the limited access to the CNS caused by protective barriers surrounding the brain such as the blood–brain barrier (BBB). These barriers pose challenges in delivering mAbs to their intended targets within the brain at concentrations necessary for their optimal efficacy. Moreover, mAbs administered directly into the cerebrospinal fluid (CSF) are rapidly cleared from the CNS to the systemic circulation, with reported half-lives from minutes to hours [1–3]. Despite these obstacles, recent FDA approvals for treatments of neurological disorders, such as Leqembi^®^ (lecanemab) and Aduhelm^®^ (aducanumab) for Alzheimer’s Disease (AD), have demonstrated the potential of mAbs for treating brain disorders. Both mAbs have shown the ability to reduce amyloid plaques in the early stage of AD [4,5]; however, the high intravenous doses of mAb required for achieving sufficient doses in the brain for its efficacy have been associated with damage to the blood–brain barrier (BBB) [6]. Therefore, many researchers are investigating new methods to safely improve the efficiency of mAb delivery to the brain. This review aims at enhancing our understanding of antibody brain exposure by investigating their uptake and clearance from the central nervous system (CNS), while also exploring the current state-of-the-art mAb delivery methods. By delving into these aspects, we strive to improve antibody brain exposure, ultimately enhancing their potential therapeutic efficacy.

### 1.1. The Blood–Brain Barrier

**Figure 1.**
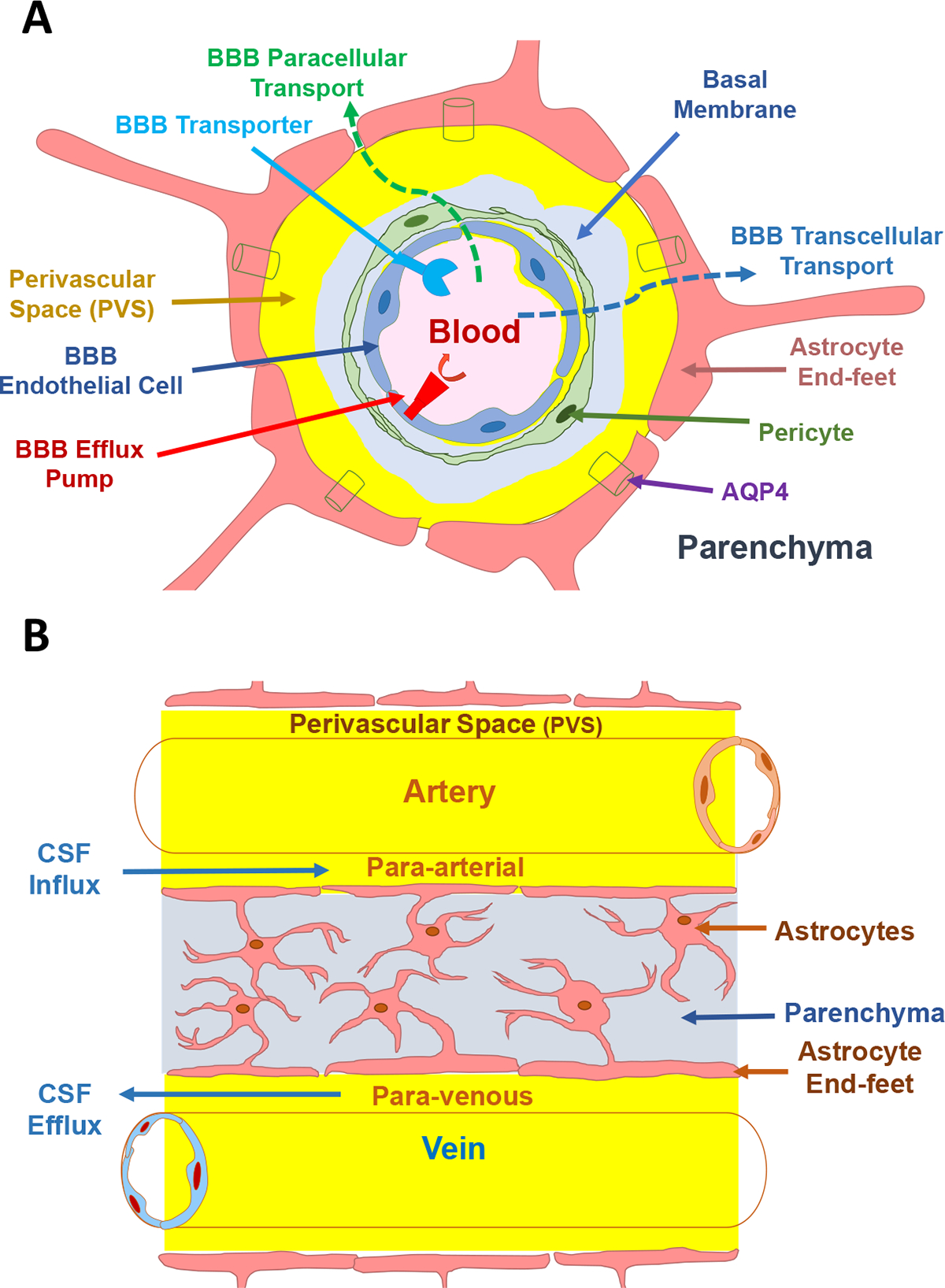
(**A**) A schematic of the BBB that is composed of endothelial cells (blue) surrounded by supportive pericytes (green) and astrocytic endfeet (pink). The basement membrane (light blue) is shared between pericytes and endothelial, while perivascular spaces (yellow) are located between the basement membrane and astrocytic endfeet and are filled with CSF. AQP4 channels on astrocytic endfeet mediate water flux into PVS. (**B**) In the glymphatic system, fluid movement is facilitated by AQP4 channels located on astrocytic endfeet, driving convective flow from the CSF-filled periarterial spaces to the perivenous spaces. This convective flow within the brain parenchyma is believed to contribute to the clearance of waste products from the brain. Subsequently, the parenchymal waste present in the CSF is drained into the peripheral lymphatics through the perivenous spaces.

The blood–brain barrier (BBB) comprises the largest interface between the central nervous system (CNS) and the systemic circulation, serving as a protective barrier that regulates the exchange of substances between the brain and the peripheral parts of the body (Figure 1A). The BBB is formed by a collaborative effort between endothelial cells, pericytes, and surrounding astrocytic endfeet, which collectively create a specialized structure known as the neurovascular unit (NVU). This unit is characterized by the close association of endothelial cells and pericytes and they share a common basement membrane [7], while being supported by the enveloping astrocytic endfeet. Together, these components contribute to the establishment and maintenance of the BBB’s selective permeability and functional integrity.

The molecular permeability of the BBB is primarily hindered by brain capillary endothelial cells (BECs), while pericytes and astrocytes in the neurovascular unit (NVU) contribute to structural stability and release chemical factors that maintain BBB integrity [7–9]. BECs impose stringent restrictions on both transcellular and paracellular transport of molecules (Figure 1A). Transcellular transport is limited due to the lack of fenestrations, abundance of mitochondria, low rates of transcytosis, and expression of efflux transporters [10,11]. Moreover, tight protein–protein interactions form tight junctions—*adherens* junctions—and desmosomes between BECs, severely impeding paracellular transport across the BBB. These BBB properties present a formidable obstacle to the delivery of therapeutic and diagnostic agents to the brain. Indeed, approximately 98% of small molecule drugs and mostly all macromolecule therapeutics are unable to penetrate the CNS through the BBB [10,12].

### 1.2. The Blood–Cerebrospinal Fluid (CSF) Barrier (BCSFB)

The choroid plexus (CP) serves as a part of a barrier known as the blood–cerebrospinal fluid (BCSFB) barrier, establishing an additional interface within the CNS for the passage of molecules. The CP is constructed of a highly vascularized stroma with specialized epithelial cells and fenestrated capillaries. Positioned strategically in each of the four ventricles of the brain, the CP plays a crucial role in CSF production. The CP actively transports ions, nutrients, and metabolism products between the blood and CSF, contributing to the maintenance of a chemically balanced environment in the CNS.

After its production by CP epithelial cells, CSF circulates from the lateral ventricles to the third ventricle through the intraventricular foramen and, subsequently, reaches the fourth ventricle via the cerebral aqueduct. From the fourth ventricle, CSF continues to circulate in the subarachnoid space lining around the brain, as well as through the spinal cord central canal. The subarachnoid space is defined by a barrier of epithelial-like arachnoid cells that separates the CSF from the fenestrated vasculature present in the dura. As a result, these arachnoid barrier cells also contribute to the BCSFB.

Similar to the BECs of the BBB, the epithelial cells of the CP and arachnoid barrier cells are joined together using tight and adherens junctions that restrict paracellular transport into the CSF. Restricted paracellular transport allows cellular transporters to control the distribution of solutes on both sides of the barrier (Figure 1A). BSCFB cells also express a wide variety of transporters, which are often distributed asymmetrically between the basolateral and apical membranes, carefully regulating chemical homeostasis [13]. Additionally, like the BECs of the BBB, CP epithelial cells contain a high number of mitochondria to meet the energetic demands of transepithelial transport [13].

## 2. Mechanisms of Antibody Uptake into the CNS

Although mAbs possess high specificity, a long systemic half-life, and minimal off-target effects, their potential as therapeutic candidates for neurological diseases is impeded by the restrictive CNS barrier. The physicochemical properties of mAbs (i.e., large size, high hydrogen bonding potential, charge) prevent them from traversing through the BBB to reach potential targets within the CNS. Nevertheless, peripheral administration of mAbs has demonstrated their presence in the CNS with CNS-to-plasma or CNS-to-serum ratios ranging from 0.1% to 0.3% [14–18]. The precise mechanisms by which mAbs in the systemic circulation achieve CNS exposure are speculative, however, several theories have been proposed.

Several mAbs have been approved for use in patients with brain diseases such as Alzheimer’s disease (AD), multiple sclerosis (MS), and brain tumors (i.e., glioblastoma, neuroblastoma) (Table 1) [19–21]. Several approved therapeutic mAbs have functions to control biological events in the peripheral tissues or outside the brain; thus, they do not need to cross the BBB into the brain for their biological activities. For example, an MS drug, Natalizumab (Tysabri), has activity to inhibit the infiltration of activated immune cells into the brain by blocking immune cell adhesion on the BBB endothelial cells [22]. The two successful mAbs (i.e., Aducanumab, Lecanemab) that target amyloid beta plaques in the brain have been approved for treating AD patients; these mAbs presumably have to cross the BBB to clear the amyloid beta plaques in the brain [4,23,24]. In contrast, several clinical trials of mAbs for the remyelination of axons in MS patients, such as VX15/2503, anti-LINGO-1 (Opicinumab), sHIgM22, and anti-Nogo-A, were terminated due to the lack of efficacy [25–28]. Similarly, the phase 2 clinical trial of anti-Tau mAb (8E12) in AD patients was stopped due to the lack of mAb efficacy [29]. In some cases, the delivery of mAb to the brain was not efficient because of the difficulty of crossing the BBB from the systemic circulation. In addition, there is still a lack of comprehensive and quantitative studies to compare the efficiency of various methods to deliver mAbs into the brain.

### 2.1. Crossing the BCSFB

To measure brain concentrations, researchers often rely on CSF concentrations to act as a surrogate for widespread brain exposures; however, doing so may produce overestimations of mAb concentrations within the brain parenchyma. Numerous studies have highlighted that molecules administered directly into the CSF experience rapid clearance and achieve minimal penetration into the brain tissue [1–3,30]. As a result, measuring antibody CSF concentrations may serve as a representation of molecular transport across the BCSFB but may not provide an accurate prediction of mAb brain deposition and therapeutic efficacy.

Evidence to support the BCSFB crossing of mAbs includes the relative “leakiness” of the BCSFB compared to the BBB. While the BCSFB and BBB have distinct permeability profiles based on specific transporter expression on their respective membranes, the BCSFB has been found to be more permeable compared to the BBB [31]. This increased permeability manifests as leakage of plasma proteins across the barrier and lower electrical resistance of the cellular barrier [31,32].

### 2.2. Non-Specific Endocytosis

In a recent study conducted by Van De Vyver et al., pharmacokinetics in the brains of healthy rats were modeled to analyze the effects of non-targeting mAbs administered via intravenous (IV) or intracerebroventricular (ICV) route [33]. Pathway analysis from their study suggested that antibody exposure in the interstitial fluid (ISF) of the brain is predominantly mediated by mAbs traversing the BBB rather than entering the ISF directly from the CSF, regardless of route of administration [33]. While some researchers have speculated that transcytosis of IgG antibodies may be facilitated by receptors on brain endothelial cells, such as the neonatal Fc receptor (FcRn), several studies have refuted this hypothesis [15,16,34]. Alternatively, other researchers have proposed that antibody uptake across the BBB occurs non-specifically via endocytic vessels in the brain [34,35].

Researchers supporting the non-specific uptake of antibodies across the BBB have highlighted that the magnitude of circulating mAb uptake into the CNS (0.1–0.3%) is comparable to other endogenous circulating proteins, such as serum albumin [35,36]. In line with this notion, several studies have reported that increasing antibody dosage leads to an increase in CNS exposure in a non-saturating fashion [34,37]. Conversely, an independent investigation examined the transport of IgG antibodies across human brain microvascular endothelial cells in an in vitro BBB model and discovered that antibody transport was saturable and reliant on macropinocytosis [36]. These findings collectively indicate that the uptake of IgG occurs through non-specific, charge-based adsorption of IgG to the negatively charged endothelial cell surface, followed by subsequent macropinocytosis. The relationship between charge and brain uptake has been demonstrated in other studies for mAbs [34,38] as well as other macromolecules such as albumin [39].

### 2.3. Antibody Clearance from the CNS

The administration of most therapeutic mAbs for neurological disorders is performed intravenously. This is because strategies to bypass the BBB through delivery directly into the CSF of the CNS have demonstrated that mAbs rapidly efflux from the CSF back into the serum with limited penetration into the brain parenchyma. This is also true for the administration of mAbs directly into the brain parenchyma, where rapid clearance half-lives have been reported and minimal diffusion throughout the whole brain tissue [40,41]. The rapid clearance of direct CNS delivery, therefore, causes these more invasive administration methods to have similar mAb exposure profiles as the IV administration. Therefore, it is imperative to improve our understanding of the potential mAb clearance mechanisms limiting their brain exposure in order to develop long-acting therapeutics for the brain.

### 2.4. Neonatal Fc Receptor

The neonatal Fc receptor (FcRn) is a class of Fc receptors recognized for its crucial role in antibody transport and recycling. FcRn facilitates passive immunity transfer from mother to young by enabling the transcytosis of IgG antibodies across the placental and intestinal mucosa barrier. The receptor is expressed on the cell surface of various cell types, including endothelial cells, epithelial cells, and antigen-presenting cells [42]. A study by Schlachetzki et al. [43] demonstrated that FcRn is expressed on the microvasculature in the brain, raising inquiries about its involvement in the transport of IgG antibodies across the BBB.

While FcRn may facilitate the bidirectional transport of antibodies across a barrier, multiple studies have found no evidence of FcRn contributing to the influx of antibodies from blood to the brain, leading to higher CNS exposure [15,16,34,44]. However, Pardridge and colleagues have suggested that FcRn may mediate brain-to-blood efflux of IgG and have demonstrated Fc-dependent elimination of IgG from the brain after intracranial administration in rats [40,45]. Similar studies by Cooper et al. observed reduced clearance of an IgG with attenuated FcRn binding following intracranial administration in rats [46]. Additionally, brain clearance of endogenous amyloid beta following intravenous administration of anti-amyloid beta (anti-Aβ) mAb was found to be reduced in *FcRn*^−*/*−^ mice [47].

While investigations by Balthasar and co-workers have challenged the idea of FcRn-mediated brain efflux, [15,16] it is important to note that study design differences may have contributed to these conflicting findings. Balthasar’s studies tracked whole brain concentrations following intravenous administration of radiolabeled mAbs in FcRn knockout mice and observed no difference in brain-to-blood AUC ratios between *FcRn*^−*/*−^ mutants and control animals [15,16]; however, whole-brain concentrations may inaccurately reflect antibody concentrations in the parenchyma, where FcRn-mediated efflux across the BBB is speculated to occur and may reflect CSF concentrations from mAb crossing the BCSFB, as discussed in previous sections.

## 3. The Glymphatic System and Bulk Convective Flow

The lymphatic vasculature plays a vital role in clearing ISF, along with its constituent proteins and solutes that are not absorbed across postcapillary venules, while also serving to maintain hydrostatic pressure [48]. This function is essential for overall tissue homeostasis. Intriguingly, despite its high metabolic rate and the remarkable sensitivity of neurons and glial cells to changes in the extracellular environment, the brain lacks a lymphatic vascular system [48]. To address this disparity, Nedergaard and colleagues proposed an alternative waste clearance system in the brain, resembling the lymphatic clearance systems in peripheral tissues [49]. They coined this system the “glymphatic system”, which serves as a mechanism for efficient waste clearance in the brain.

The glymphatic system is connected to perivascular space (PVS) and aquaporin-4 (AQP4) and it operates by utilizing the transport of CSF in the perivascular spaces (PVSs) of the brain (Figure 1B). The pial of the artery in parenchyma is connected to the Virchow-Robin spaces (VRS) that surround the arteries, venules, and capillaries to form donut-like tunnel spaces called PVS (Figure 1B) [50]. PVS is constructed by a combination of smooth muscle and vascular endothelial cells at the inner wall while the outer wall is constructed of the astrocyte endfeet. Arterioles that penetrate the brain parenchyma contain PSV which are finally fused basal lamina containing extracellular matrix proteins (ECM), including laminin, fibronectin, and collagen. This allows the CSF to influx along the peri-arterial space. In this case, CSF can enter the brain parenchyma via PVS to mix with ISF for delivering nutrients or clearing metabolites.

The glymphatic system delivers nutrients to the parenchyma via periarterial CSF influx as well as removes metabolism waste via perivenous routes for clearance of cell debris and unwanted large metabolites (i.e., proteins) using AQP4 on astrocytes. These regions consist of CSF-filled spaces between the basement membranes of brain endothelial cells and the astrocytic endfeet (Figure 1A). The proposed pathway for fluid flow and waste removal begins with CSF from the subarachnoid space moving along periarterial spaces into the brain (Figure 1B). CSF then leaves the periarterial spaces to mix with ISF within the brain parenchyma before being transported via convective flow to perivenous spaces (Figure 1B). The CSF in perivenous spaces will then drain into the peripheral lymphatic system. The functionality of this system relies on the continuous flow of fluid through the brain tissue extracellular space with the help of aquaporin-4 (AQP4) on the astrocytic endfeet near the basement membrane of brain endothelial cells (Figure 1A). The bulk flow of convective movement propels fluid through the brain parenchyma, aiding in the clearance of waste products into the CSF-filled perivenous spaces for eventual peripheral lymphatic clearance.

Nedergaard’s key experiments that contributed to the discovery of this system involved tracking the movement of fluorescently or radioactively labeled tracer molecules with various molecular weights following intraparenchymal and intracisternal administration. After intracisternal injection, they observed the CSF movement in perivascular spaces and in the ESC of the parenchyma characteristic of the glymphatic system described above [49]. Notably, molecules of significant molecular weight differences cleared from the parenchyma at similar rates, indicating that convective bulk flow, rather than diffusion, is responsible for their clearance [49]. Additional studies have also provided support for glymphatic clearance mechanisms [49,51–55]. The proposed bulk convective flow within the ECS, as suggested by the glymphatic system, presents a potential mechanism for antibody clearance after distribution in the parenchyma.

## 4. Diffusion

The proposed concept of convective flow facilitating clearance from the brain parenchyma, as suggested by the glymphatic system, faces challenges from several researchers who contend that molecular transport in the extracellular space (ECS) of the parenchyma is primarily driven by diffusion. These researchers argue that the ECS of the brain parenchyma is intricately structured, and characterized by the presence of numerous cell bodies and processes with diverse sizes and shapes [56]. Additionally, the ECS is composed of a complex solution of proteins and glycosaminoglycans, imparting gel-like properties to the fluid [57]. Consequently, these factors lead researchers to assert that convective flow, facilitated by AQP4, would not be adequate to overcome the substantial hydraulic resistance exhibited by the brain.

Numerous studies provide evidence for the diffusive transfer of solutes through the ECS of the brain parenchyma. Contrary to the findings of Iliff et al. [49] for supporting the glymphatic system, experiments conducted in Verkman’s laboratory had demonstrated that the transfer of fluorescent dextrans in the brain parenchyma was size-dependent and not dependent on cardiorespiratory rate or AQP4-gene deletion [58]. Similar studies by Pizzo and colleagues utilized the same infusion rate/site and duration as the key experiments that established the concepts of the glymphatic system [49,59]. However, they found that diffusion was the predominant transport process governing distribution into the ECS following administration into the CSF [59]. If diffusion is indeed the primary mechanism responsible for waste clearance from the gel-like ECS, the clearance rate of antibodies would be affected by a gel filtration effect influenced by factors such as molecular shape, charge, and size.

## 5. Antibody Delivery Strategies into the CNS

A comprehensive understanding of the mechanisms governing mAb uptake and clearance in the brain is pivotal for researchers seeking to optimize delivery and retention strategies, ultimately maximizing exposure profiles of mAb in the brain. Many researchers concentrate their efforts on enhancing the permeation of mAbs across the BBB. These efforts include enhancing mAb delivery through transcellular pathways—often utilizing receptor-mediated transcytosis (RMT) delivery—or paracellular pathways by disrupting the tight junction proteins that bind these BBB cells together (Figure 1A). Additionally, novel approaches are being explored to promote mAb retention, allowing for gradual accumulation within the brain over time. While no delivery method has been used to achieve FDA approval of antibodies to date, several strategies have demonstrated remarkable potential. The continued development of these strategies may hold significant implications for the treatment of neurological disorders.

### 5.1. Receptor-Mediated Transcytosis (Trojan Horse Method)

Some circulating endogenous proteins are capable of traversing the BBB through specific receptor transporters on the endothelial cells of the BBB; such as transferrin, insulin, and leptin [60]. The discovery of these receptor-mediated transport (RMT) systems has led to so-called “Trojan Horse” delivery systems for antibodies, where antibodies are genetically modified to bind to an RMT system to induce transfer across the BBB [60]. Initial studies proved that antibodies targeting the transferrin receptor in rats [61,62], or the human insulin receptor [63], demonstrated the ability to undergo RMT to increase brain exposure.

Antibodies directed against human insulin receptors (HIRs) or transferrin receptors (TfRs) have been effectively employed as antibody drug conjugates (ADCs) to facilitate the transport of smaller peptides or proteins across the highly restrictive BBB. In one study, a vasoactive intestinal peptide (VIP) was conjugated to an anti-transferrin mAb (OX-26) via an avidin–biotin linkage [64]. When applied topically to brain surface vessels, VIP alone is a potent cerebral vasodilator but it is incapable of crossing the BBB independently. However, the infusion of the OX-26-VIP conjugate in rats resulted in a significant 65% increase in cerebral blood flow compared to the controls of OX-36 or VIP administered alone [64]. Another study employed chemical conjugation to link nerve growth factor (NGF) to OX-26, which, when tested in an extra-cranial anterior eye transplant model, exhibited enhanced survival rates of both cholinergic and non-cholinergic neurons compared to unconjugated OX-26 and NGF controls [65]. Additionally, ADCs employing RMT mAbs have been utilized for diagnostic purposes in a primate study to examine amyloid levels in the brain [66].

Bispecific antibodies targeting the TfR or HIR have also been developed to increase antibody-BBB penetration and exert potential therapeutic effects. The first antibody engineered of this kind was tetravalent, wherein the carboxyl terminus of a bivalent genetically engineered antibody against the human insulin receptor (HIR) was fused with two anti-amyloid β (anti-Aβ) single-chain variable fragments (ScFv) [45]. However, several studies have indicated that increasing the affinity/avidity of antibodies against transferrin or insulin leads to substantial accumulation and degradation within brain capillary endothelial cells (BCECs), with limited transport into the brain tissue beyond the capillaries [67–69]. Therefore, Yu et al. investigated the correlation between TfR affinity and brain uptake and were the first to examine monovalent bispecific antibodies targeting the TfR [70]. Their research revealed that decreasing the TfR affinity resulted in an increase in brain exposure. They also demonstrated the increase in BBB transport of a genetically engineered bispecific antibody targeting TfR and β-secretase (BACE-1) compared to that of a monospecific anti-BACE-1 mAb [70]. BACE-1 is an enzyme important for processing Aβ peptides associated with AD. Supporting these findings, monovalent binding of mAb to TfR increased transport across the BBB compared to bivalent TfR binding; this monovalent binding influenced lysosome sorting of the mAb [71].

TfR mAbs and HIR mAbs face several safety concerns for their development. While these antibodies are developed to target epitopes on receptors separate from iron or insulin binding locations, [72] they possess the potential for both agonistic and antagonistic effects on the receptors. For example, hypoglycemia was observed in primates who received high doses of HIRmAb-IDUA (human α-L-iduronidase) [73]; however, no such effect was observed at low doses or when infused in humans [74]. Additionally, studies have indicated that TfR mAbs may lower iron uptake, either through antagonist effects or through depletion of TfR on cell membranes [75].

These promising findings have sparked hope for the development of effective treatments for CNS diseases, particularly mucopolysaccharidosis type II [76–78] and AD [79]. With the ongoing clinical trials for RG6102 as a novel antibody therapeutic for AD, the ability of the RMT brain shuttle technology to facilitate the crossing of the blood–brain barrier and target amyloid plaques in AD mouse models has been demonstrated [71,80]. These advancements hold great potential for the future of CNS disease treatment, paving the way for improved patient outcomes and enhanced quality of life.

### 5.2. Osmotic BBB Opening

Experiments in the 1970s demonstrated the reversible opening of the BBB in rabbits through intracarotid administrations of hyperosmolar concentrated electrolyte solutions [81,82]. It is believed that the reversible opening of the paracellular pathways (Figure 1A) of the BBB was a result of the osmotic withdrawal of water from BBB endothelial cells, causing cell shrinkage and tight junction separation. The increase in molecular permeability to the brain via the paracellular pathways of the BBB has been shown to be size-dependent, with higher permeability of small molecules compared to larger molecules [83]. In addition, the osmotic BBB opening (OBBBO) or BBB disruption (BBBD) method has demonstrated the ability to increase the delivery of proteins with large molecular weights, including albumin, antibodies such as Fab fragments, immunoglobulin G (IgG), and immunoglobulin M (IgM) [84–92]. Following BBBD, an increase in the CNS concentration of endogenous neutralizing IgG was observed in primates that were immunized against measles; this result demonstrates the potential of BBBD to improve efficacy of immunotherapy in treating infections in the brain [87]. Additionally, exogenous IgG delivery was increased with BBBD following intravenous administration of the mAb in rats [86].

There are some safety concerns that have been observed with the BBBD method. BBBD produces a long-term opening of the BBB to allow circulating large molecules (e.g., albumin and fibrinogen) to enter the brain and produce toxic effects in the brain tissues [93,94]. One study found that the nerve damage caused by the uptake of these plasma proteins into the brain may be irreversible [95]. Preclinical studies in rats have demonstrated that BBBD induces microglial activation and a sterile inflammatory response in the brain [96], and alters cerebral blood flow [97]. Additionally, clinical studies using BBBD to deliver oncology agents to patients found a 13% incidence of seizures associated with the delivery method [95].

### 5.3. Focused Ultrasound Microbubbles

Focused ultrasound (FUS) is a technique that utilizes acoustic energy to increase BBB permeability in focal regions of the brain. This method was developed based on the findings that ultrasound waves can cause cavitation and collapse of tiny gas-filled bubbles in fluids. In tissues, focused ultrasound (FUS) can cause cavitation within blood vessels, which can cause various effects based on the frequency and intensity of radiation. The effects of FUS on the brain have been studied since the 1960s, where the high frequencies used could induce the BBB opening but also resulted in lesions within the parenchyma [98]. In 1995, studies were conducted to refine the sonication parameters to induce BBB opening while minimizing tissue damage [99]. It was later discovered that combining FUS with IV administration of tiny gaseous microbubbles (MB) can drastically lower the acoustic parameters needed to facilitate BBB opening without damaging the tissue [100]. MB help reduce acoustic parameters by acting as cavitation sites under FUS, where the mechanical forces disrupt endothelial cells of the BBB to allow molecules to enter the brain.

Several studies have investigated the size of the BBB opening from FUS-MB using fluorescently labeled dextran molecules [101,102]. A constant acoustic pressure of 0.57 MPa delivered 3 kDa and 70 kDa dextran molecules across the BBB but not 2000 kDa dextran [101]. A parallel study found that increasing the acoustic pressure to 0.84 MPa could facilitate the delivery of 2000 kDa dextran [102]; however, pressure at 0.84 MPa was found to induce inertial cavitation and cause microhemorrhages in the brain [102]. When examining the brain permeability of liposomes of sizes ranging from 55 to 200 nm using FUS-MB, increasing the MB dose from 0.1 μL/g to 0.5 μL/g significantly enhanced the delivery of the 200 nm liposomes. These results indicate that the extent of BBB opening with FUS-MB is determined by both the acoustic parameters and the injected dose (ID) of MB.

A combination of modulating the ID of MB and acoustic parameters has allowed researchers to deliver mAbs to the brain using FUS-MB. Numerous preclinical studies in mice demonstrate the ability of FUS-MB to effectively deliver antibodies to the brain with minimal tissue damage, including an anti-HER2 mAb [103] and a mAb against the dopamine D4 receptor [104]. Antibody delivery with FUS-MB has shown promise in enhancing the therapeutic efficacy of mAbs for brain diseases. For example, FUS-MB resulted in a 5.5-fold increase in delivery of anti-pyroglutamate-3 anti-Aβ mAb to the brain of APP/PS1dE9 mice, a model for AD, and improved spatial learning and memory in the animals at a faster rate [105]. In a xenograft mouse model of high-grade glioma from a patient, FUS-MB increased the delivery of a tumor-targeting antibody to localized tumor regions of the brain. Due to the success of preclinical studies, several clinical safety studies have been evaluated in humans for brain tumors [106] and AD [107]. These clinical safety studies have demonstrated FUS-MB to be a well-tolerated potential delivery method in humans, making this a promising approach for treating CNS disorders with mAbs.

### 5.4. BBB-Modulating Peptides

One approach to improve the paracellular permeability of the BBB is by disrupting protein–protein interactions between the BBB endothelial cells using small cadherin-derived BBB modulator (BBBM) peptides. These peptides block cadherin–cadherin interactions, temporarily increasing the porosity of the BBB to enable the transport of molecules from the blood into the brain. In vitro studies have demonstrated BBBM activity of these peptides by inhibiting calcium-dependent reaggregation [108], lowering transepithelial electrical resistance (TEER) [109,110], and increasing paracellular transport of ^14^C-mannitol in tight junction-forming cell monolayers [110]. Additionally, BBBMs in animal models (mice and rats) have demonstrated increased in vivo brain delivery of small molecules (i.e., anticancer agents [111,112], mannitol [111,113], gadopentetic acid [113–115]), medium-sized peptides [116], and large molecules, including Brain-Derived Neurotrophic Factor (BDNF) (13 kDa) [117,118], IRDye800CW PEG (25 kDa) [114,115], albumin (67 kDa) [116,119], and IgG mAb (150 kDa) [119,120]. Successful in vivo delivery of molecules has been detected with Magnetic Resonance Imaging (MRI), Near-IR Fluorescence (NIRF) imaging, mass spectrometry, and radioactivity counts.

Co-dosage of an IgG mAb with BBBM peptides in mice resulted in a significant 2–4-fold increase in mAb deposition within the brain compared to the administration of mAb alone [119,120]. The extent of increase varied depending on the specific BBBM peptide used, with cyclic BBBM peptides demonstrating higher enhancement compared to linear counterparts [120]. Multiple injections of BBBM after one administration of mAb can significantly enhance mAb brain deposition compared to only one administration of BBBM along with mAb. While studies have demonstrated the improved therapeutic efficacy of BDNF through enhanced brain deposition using BBBM peptides in mouse models of multiple sclerosis (MS) and Alzheimer’s disease (AD) [117,118], no investigations have been conducted to determine the therapeutic impact of enhanced antibody brain deposition achieved with these peptides.

To date, no safety concerns have been reported with the BBBM peptides. Repeat administration of peptides in mice did not result in weight loss or change in locomotive activity [121]. In addition, this study also showed no astrogliosis and inflammation in the brains of mice treated with BBBM [121]. Unlike other methods such as BBBD, BBBM enhanced paracellular permeability and did not alter cerebral blood flow [114]. Notably, although BBBMs have the ability to increase albumin brain deposition [116,119], which can be toxic to the brain [93], minimal toxicity has been observed. One potential reason for the minimal toxicity is that the opening of the BBB by BBBM peptides is transient and reversible. The BBB opening was observed between 1 and 4 h for a small molecule [113,114] (Gd-DTPA, MW ~500 Da) and less than 40 min for a large molecule (Galbumin, MW ~65 kDa) [116]. Furthermore, BBBMs create the opening with a molecular size limit; they enhance the permeation of 150 kDa IgG mAb across the BBB but not 220 kDa fibronectin [119]. The duration time of the BBB opening by BBBM was short and the brain deposition was dependent on the size of the delivered molecule. Therefore, it limits the penetration of unwanted proteins in the blood from entering the brain. However, further safety assessment may be necessary through dose escalation studies conducted over extended periods of time. Overall, these findings highlight the potential of BBBM peptides as a promising strategy for enhancing the delivery of therapeutic agents across the BBB; however, further research is needed to fully evaluate their therapeutic impact and long-term safety.

### 5.5. Enhancing Antibody Retention

IgG mAbs have been observed to undergo rapid efflux after delivery into the CNS [1–3]. To increase the brain exposure of mAbs, one potential approach is to improve their retention once delivered. Although there has been limited scientific exploration of brain retention approaches for mAbs, one group has demonstrated that mAb binding to neural molecules within the CNS matrix can significantly enhance brain exposure [122]. In this study, the brain exposure of anti-MOG mAb targeting myelin oligodendrocyte glycoprotein (MOG), which is a brain-specific target, was compared to anti-TfR mAbs (known to cross the BBB through RMT) and a non-targeting control mAb. While the anti-TfR mAb exhibited higher brain concentrations at shorter time periods (4 days), the anti-MOG mAb demonstrated a significantly higher brain concentration as well as a longer brain exposure (10 days) compared to control mAbs. This finding can be explained by the fact that while the anti-TfR mAb rapidly accumulates in the brain, it is rapidly cleared from systemic circulation due to the widespread distribution of TfR throughout the body. In contrast, the anti-MOG mAb does not accumulate rapidly in the brain due to the restrictive CNS barriers. However, it has a much longer plasma half-life and binds strongly to its neuronal target due to its retention upon binding to target MOG protein in the brain. This target engagement allows the small amounts of mAb that access the brain from the systemic circulation to evade efflux and accumulate over time. These results highlight the potential for enhancing brain exposure by increasing mAb retention as a promising avenue for mAb treatment of brain disorders.

### 5.6. Brain Delivery Using Nanoparticles

Various nanoparticles have been developed for brain delivery of small drugs and macromolecules (e.g., mAbs) [123–125], including extracellular vesicles (EVs) [126], solid lipid nanoparticles [127], exosomes [128–130], nanobubbles [131,132], nanocages [133], leukocyte biomimetic nanoparticles [124], and recombinant adeno-associated viruses (rAAVs) [134–136]; however, these methods have not yet been successfully utilized in the clinic for delivering mAbs into the brain. Nonetheless, these methods could help in delivering mAbs as therapeutic and diagnostic agents for brain diseases.

### 5.7. Intranasal Brain Delivery of Proteins and Peptides

Intranasal brain delivery method has been explored to deliver peptides (e.g., oxytocin) and proteins (e.g., insulin) to the brain and some of them have reached clinical trials for treating neurodegenerative diseases (e.g., Alzheimer’s disease, autism spectrum disorder (ASD)) [137]. Nasal delivery of peptides has been shown to be more effective than IV and intraperitoneal (IP) administrations because it avoids peptide degradation in the blood [138–140]. The delivered molecules have to cross the nasal epithelial layer and cribriform plate for diffusion to the olfactory bulb as well as the trigeminal nerve; thus, several intranasal delivery enhancers were investigated such as tight junction disruptors (i.e., carnitines and ultrasound), CPP, receptor-mediated transport, and nanoparticles [138,139]. Although some of these methods have been approved or are undergoing clinical trials, many of these are still under investigation in the preclinical setting. If successful, these methods will help to treat patients with CNS disorders.

## 6. Conclusions

The progress in developing mAb therapeutics for brain disorders has been slow due to their limited CNS exposure and potential safety concerns with increasing permeation across CNS barriers. Understanding the potential mAb uptake mechanisms can give insight into potential delivery strategies for increasing mAb penetration across the BBB. Many methods have focused on this idea and have shown promise in safely facilitating this delivery. Additionally, knowledge of mAb clearance from the brain also aids in efforts to improve the exposure of antibodies. While many methods have been explored and shown promise for enhancing mAb brain exposure, there has been limited success in utilizing these methods in patients with CNS diseases that resulted in FDA approvals. Therefore, there is still a current need for expanding research and development in this research area.

## Figures and Tables

**Table 1.** Monoclonal Antibodies for CNS Diseases.

Alzheimer’s Disease			
Name (Brand)	Target	mAb Type	US Approval (Status)
Aducanumab (Aduhelm)	Amyloid beta	Human IgG1	2021
Lecanemab (Leqembi)	Amyloid beta	Humanized IgG1	2023
Donanemab	Amyloid beta	Humanized IgG1	2nd Review
LY3372993; Remternetug	Amyloid beta	Human IgG1	Phase 3
Crenezumab	Amyloid beta	Humanized IgG4	Phase 3
Gantenerumab	Amyloid beta	Human IgG1	Phase 3
Solanezumab	Monomers	Humanized IgG1	Phase 3
E2814	Tau protein	Humanized IgG1	Phase 2/3
Semorinemab	Tau protein	Humanized IgG4	Phase 2
BIB092	Tau protein	Human mAb	Phase 2
ABBV-8E12	Tau protein	Human mAb	Phase 2
Zagotenemab	Tau protein	Human mAb	Phase 2
JNJ-63733657	Tau protein	Human mAb	Phase 1
AL002	TREM-2 Receptor	Human mAb	Phase 1
AL003	SIGLEC-3	Human mAb	Phase 1
**Frontotemporal Dementia**			
AL001; Latozinemab	Sortilin	Human IgG1	Phase 3
**Glioblastoma**			
^125^I-mAb 425	EGFR	Human mAb	Phase 2
Depatuxizumab mafodotin	EGFR	IgG1 ADC	Phase 2b/3
[^188^Re]-labeled Nimotuzumab	EGFR	Humanized mAb	Phase 1
^131^I-chTNT-1/B MAb	DNA-histone H1 complex	Human mAb	Phase 1/2
^131^I-BC-2 mAb	Tenascin	Human mAb	Phase 2
^211^At-labeled 81C6 mAb	Tenascin	Human mAb	Phase 1/2
biotin-coupled BC-4 + Avidin + [90Y]-Biotin	Tenascin	Human mAb	Phase 1/2
**Neuroblastoma**			
Dinutuximab (Unituxin)	GD2	Chimeric IgG1	2015
^131^I-omburtamab	BT-H3	Murine mAb	Phase 2/3
**Multiple Sclerosis (MS)**			
Daclizumab (Zinbryta)	CD25	Humanized IgG1	2016
Divozilimab (Ivlizi)	CD20	Humanized IgG1	2023
Ocrelizumab (OCREVU)	CD20	Humanized IgG1	2017
Ublituximab (BRIUMVI)	CD20	Humanized IgG1	2022
Alemtuzumab (Lemtrada)	CD52	Humanized IgG1	2014
Natalizumab (Tysabri)	α4 integrin	Humanized IgG4	2014

Adapted from Refs. [19–21].
